# The PEA Bead Test as a Screening Tool for Olfactory Dysfunction: A Preliminary Study

**DOI:** 10.3390/life13102074

**Published:** 2023-10-17

**Authors:** Kyung Soo Kim, Il-Youp Kwak, Hyun Jin Min

**Affiliations:** 1 Department of Otorhinolaryngology-Head and Neck Surgery, College of Medicine, Chung-Ang University, Seoul 06973, Republic of Korea; 2 Department of Applied Statistics, Chung-Ang University, 224-1 Heukseok-dong, Dongjak-gu, Seoul 06973, Republic of Korea; ikwak2@cau.ac.kr

**Keywords:** olfaction, smell, anosmia, screening

## Abstract

This study, conducted in a single tertiary hospital, aimed to evaluate the 2-phenylethyl alcohol (PEA)-based sniffing bead test as a screening tool for olfactory dysfunction suitable for implementation in otorhinolaryngology and other settings, including general practice clinics. A total of 139 patients were enrolled, all of whom underwent both the PEA bead test and conventional psychophysical olfactory function testing. Characteristics such as age, sex, concurrent diseases, smoking history, and presence of gustatory dysfunction were reviewed. Statistical analyses included receiver operating characteristic curve analysis, area under the curve determination, and multivariate linear regression guided by the Akaike information criterion. Significant correlations were found between the PEA bead test and various subsets of the conventional YSK olfactory function test: threshold (r = 0.631), discrimination (r = 0.455), and identification (r = 0.596), as well as the composite threshold-discrimination-identification score (r = 0.686). These correlations remained significant even when adjusting for other clinical characteristics. The PEA bead test showed a sensitivity of 0.778 and a specificity of 0.958 for discriminating olfactory dysfunction at a cutoff value of ≥1. These findings indicate that the PEA bead test could be a valuable screening tool for olfactory dysfunction across diverse clinical settings. Further large-scale, multicenter research is recommended to confirm these preliminary results.

## 1. Introduction

Anosmia has a reported prevalence of about 0.8% to 5.8% in the general population [1]. Impaired olfaction may occur as part of the natural aging process, after viral infection, or after head trauma. Olfactory dysfunction could also accompany various conditions, including neurodegenerative diseases, such as Alzheimer’s disease or Parkinson’s disease, diabetes, obesity, autoimmune diseases, and mental illnesses [2,3]. Notably, during the COVID-19 pandemic, olfactory dysfunction was commonly encountered as a symptom of SARS-CoV-2 infection, and the significance of appropriate diagnoses in this context is underscored by the high prevalence of this symptom [4].

Various olfactory function tests have been developed. For example, culture-specific psychophysical assessment tools, such as Sniffin’ Sticks, have been traditionally developed and used to evaluate olfactory function [5,6,7]. Subjective olfactory evaluation based on questionnaires has been validated and actively applied to COVID-19 patients [8,9]. Recently, a more objective indicator—olfactory event-related potentials—has been introduced, although this measure is not widely used [10].

Psychophysical methods for assessing olfactory function, which have long been in use and are commonly applied, enable quantitative measurements and can be modified using culture-specific odorants. A comprehensive psychophysical olfactory function test is based on the Sniffin’ Sticks test, which comprises tests of odor threshold, odor discrimination, and odor identification [11]. As the Sniffin’ Sticks test has long been applied, studies about the results of each test subset and their clinical significance have been reported. For example, it is commonly acknowledged that the detection threshold, discrimination, and odor identification are influenced by the early, intermediate, and advanced stages of olfactory processing, respectively, necessitating varying degrees of cognitive involvement [5,12]. Patients with olfactory dysfunction due to sinonasal diseases could be particularly impaired in their odor threshold scores, whereas patients with Parkinson’s disease could show impairments specifically in the odor discrimination and identification domains, although olfaction could be individually influenced by various factors, such as patient sex and genetic aspects [13]. Odor identification largely depends on patients’ prior exposure to culture-specific odor stimuli [14]. Sniffin’ Sticks-based psychophysical assessment tools have been modified and validated in several countries throughout the world [11]. In the Republic of Korea, a Sniffin’ Sticks test consisting of threshold, discrimination, and identification test subsets that uses culturally familiar odorants has been developed, validated, and widely applied in ear, nose, and throat (ENT) departments [5]. Although this test is useful, it is time-consuming and requires patients to concentrate throughout its duration, and these are major hurdles in completing the test, especially for older people. Furthermore, the test’s application is limited when patients are suspected of having acute respiratory infectious diseases, such as COVID-19, because it is designed for multiple usage.

Therefore, we tried to evaluate much simpler, subject-friendly olfactory screening tools that could be more widely applied. We previously developed a sniffing bead system that was originally developed for evaluating olfactory function in older adults [15]. In this study, we extended our previous research and tried to evaluate the usefulness of the sniffing bead system as an effective olfactory dysfunction screening tool.

## 2. Materials and Methods

### 2.1. Study Design

This was a single-center study at a tertiary hospital, and the study protocol was approved by Chung-Ang University Hospital’s institutional review board. We enrolled patients who presented with subjective olfactory or gustatory dysfunction and performed both the sniffing bead test and the YSK olfactory function test (YOF) (RHICO Medical Co., Seoul, Republic of Korea) between October 2021 and December 2022. Patients who had been previously diagnosed with Parkinson’s disease, Alzheimer’s disease, or cognitive impairment were excluded, as were those who had been diagnosed with chronic rhinosinusitis with nasal polyps, those who had taken oral or systemic steroids within 2 weeks before screening, and those who needed surgical treatment. Data on demographic and clinical characteristics, including age, sex, and smoking status, were reviewed.

### 2.2. Sniffing Bead Test

A previously developed sniffing bead system was used [15]. The sniffing bead system consists of odorant beads, a handpiece, and a lid (Supplementary Figure S1A). The core material is 2-phenylethyl alcohol (PEA), and surfactants, sodium alginate, and safranine pigment are packaged into a gel-type sphere (diameter 4.0 ± 1.0 mm, weight 0.03 ± 0.01 g). A disposable plastic capsule container made from polypropylene is to be mounted on an aluminum-made body. The capsule container’s lid is equipped with a pointed, sharp, pin-shaped component. If the subject closes the capsule container’s lid, the sharp pin-shaped component reaches the odorant bead. The odorant beads are soft, with a gel-like consistency. The beads are to be placed in the capsule container and can be jabbed with the lid’s pin-shaped component. After the bead is punctured by the pin-shaped portion, the fragrance can be released from the odorant bead [15]. The odorant beads are designed for single use only, and the capsule container can be easily separated from the body by turning it. The body can be wrapped with vinyl before use. In this study, we used PEA, which has been used in the YOF test, as an odorant. Odorant beads were packed with various PEA concentrations (from 10% to 0.078%), and beads packed with distilled water were used as negative controls (Supplementary Figure S1B). The sniffing bead test was performed according to a previously described protocol [15]. We chose certain concentrations of PEA beads for the first trial, and beads were ruptured 2 cm anteriorly to the nostrils in the midline. If the patient recognized the odorant, the procedure was repeated with a lower concentration of odorant beads. The lowest concentrations at which the participants detected the PEA were scored as the respective PEA threshold scores (scores: 1 (highest concentration) to 8 (lowest concentration), and 0 (not detected)).

### 2.3. YOF Test

The YOF test is a validated conventional psychophysical olfactory function test, which is composed of 3 subsets (threshold, discrimination, and identification) [5]. The YOF test was performed according to the manufacturer’s instructions [5]. Briefly, the detection threshold was obtained as the concentration at which PEA was correctly identified 4 consecutive times (highest concentration, 10%; 1:2 serial dilutions to 12 steps). The discrimination score for each participant was the sum of the number of correct answers when selecting the unique odorant among 3 odorants (2 identical, 1 different). The identification score for each participant was determined as the number of correct answers based on multiple forced choices from 4 descriptors. The threshold-discrimination-identification (TDI) score for each participant was the sum of the threshold, discrimination, and identification scores. Possible TDI scores ranged from 1 to 36, and the participants’ olfactory function outcomes were labeled as normosmia, hyposmia, or anosmia based on previously reported criteria [5]. All olfactory function tests were performed by the same skilled technician in a well-ventilated laboratory.

### 2.4. Statistical Analysis

Descriptive statistics for continuous and categorical variables are presented as mean ± standard deviation and frequency (%), respectively. The characteristics of the participants who were enrolled in the study groups (categorized based on the aforementioned olfactory function labels) were compared using analysis of variance (ANOVA), chi-square analysis, and Fisher’s exact test, as deemed appropriate. ANOVA was employed to assess statistically significant differences among groups for continuous variables, such as age. For categorical variables, the chi-square test was used, with Fisher’s exact test performed when any cell had a count of 5 or fewer [16]. To demonstrate the usefulness of the PEA sniffing bead test, we used two binary classifiers. The first classifier discriminated the combined anosmia-hyposmia group from the normosmia group, and the second classifier differentiated the anosmia group from the combined hyposmia-normosmia group. To measure the performance of these two binary classifiers, we used receiver operating characteristic (ROC) curve analysis and area under the ROC curve (AUC) values [17]. ROC curves are visual representations of the diagnostic capabilities of binary classifier systems. They depict the true-positive rate (TPR, sensitivity) vs. the false-positive rate (FPR, specificity) at different threshold levels. The AUC is a summary statistic representing the performance of the binary classifier. A contour plot visually represents a 3D surface by plotting constant Z-level lines (contours) on a 2D plane. A kernel density estimation is used to estimate the 3D surface. Tighter lines on such plots signify quicker changes in the Z variable with variations in the X and Y variables. Multivariate linear regression models were fitted to explanatory variables, age, sex, diabetes mellitus status, hypertension status, smoking history, gustatory dysfunction status, and PEA sniffing bead test results. Stepwise selection with the Akaike information criterion was performed to derive the final multivariate linear regression model. To evaluate the performance of the multivariate linear models, we randomly divided our dataset into 7 and 3 training and test datasets, respectively. Models were trained on the training dataset, and the evaluation was conducted on the test dataset using ROC curves and AUC values. DeLong’s test was used to evaluate for statistically significant differences between the 2 AUCs of the 2 models. Statistical analyses were performed using R, version 4.2.2 (R Foundation for Statistical Computing, Vienna, Austria).

## 3. Results

The demographic and clinical characteristics of the enrolled participants are summarized in Table 1. Of the 139 participants, 35 had anosmia, 26 had hyposmia, and 78 were normosmic. The mean ages were 52.71 ± 18.8 years in the anosmia group, 56.62 ± 17.95 years in the hyposmia group, and 43.4 ± 17.69 years in the normosmia group (*p* = 0.004). There were no significant intergroup differences in sex composition, diabetes mellitus prevalence, hypertension prevalence, or smoking status. The prevalence of gustatory dysfunction was 51% in the anosmia group, 31% in the hyposmia group, and 10% in the normosmia group, with a statistically significant difference between the normosmia group and the others (*p* < 0.001) (Table 1).

**Figure 1:**
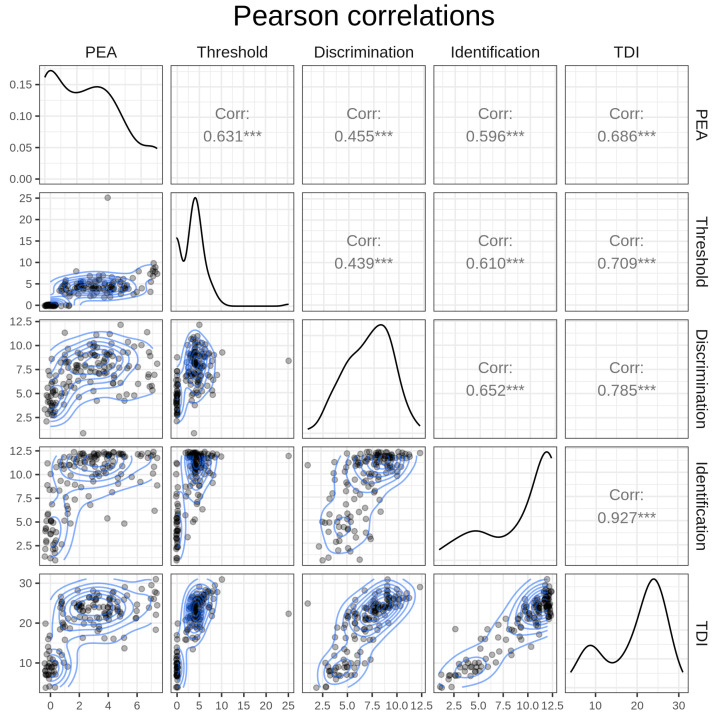
Correlation between the PEA bead test and conventional olfactory function testing. Top-right triangular components represent Pearson correlations, bottom-left triangular components depict two-dimensional density mapping with jittered data, and diagonal components represent variable density using kernel density estimation. A contour plot visually represents a 3D surface by plotting constant Z-level lines (contours) on a 2D plane. *** *p* ≤ 0.001.

The diagonal components of Figure 1 show the density of each variable, the top-right triangular components represent Pearson correlations, and the bottom-left triangular components depict two-dimensional density. The threshold, discrimination, and identification subscores had high Pearson correlation values (r) of 0.701 to 0.927 with the TDI score variable. The results of the PEA bead test significantly correlated with the threshold (r = 0.631), discrimination (r = 0.455), identification (r = 0.596), and TDI subscores (r = 0.686) of the YOF test (Figure 1).

Next, we evaluated the relationship between the PEA bead test outcomes and YOF test outcomes with consideration of other variables. Table 2 presents four fitted linear models for the YOF test results. In all four models, the PEA test results and the presence of gustatory dysfunction were selected as significant variables. The coefficient of determination (R^2^) was highest for the TDI model (R^2^ = 0.55), followed by 0.39, 0.34, and 0.42 for the threshold, discrimination, and identification models, respectively, suggesting that the PEA bead test results were well fitted with the YOF test TDI scores (Table 2).

**Figure 2:**
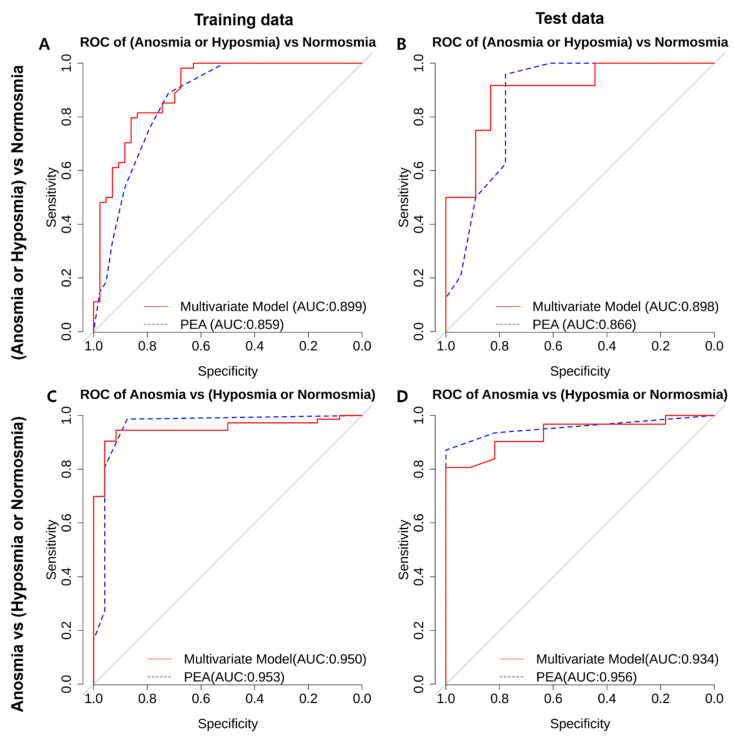
ROC curve analysis of PEA for discrimination of olfactory dysfunction. (**A**,**B**) ROC curves for the discrimination of the combined anosmia-hyposmia group vs. the normosmia group using the multivariate regression model (red line) and the PEA score-only model (blue dotted line) with the (**A**) training dataset and (**B**) test dataset. The AUC values for each case are also presented. (**C**,**D**) ROC curves for the discrimination of the anosmia group from the combined hyposmia-normosmia group using the multivariate regression model (red line) and the PEA score-only model (blue dotted line) with the (**C**) training dataset and (**D**) test dataset. The AUC values for each case are also presented.

We further evaluated the ROC curves and AUC values for discriminating olfactory dysfunction using a multivariate regression model. The combined anosmia-hyposmia group was compared with the normosmia group using the multivariate regression model considering the PEA bead test results and other clinical characteristics (red line), as well as the PEA bead test results only (blue dotted line) for the training and test datasets. The multivariate regression model performed slightly better, with AUCs of 0.988 and 0.898, while the PEA bead test-only model achieved AUCs of 0.859 and 0.866 with the training and test datasets, respectively (Figure 2A,B). The difference between the two AUCs in the training data was statistically significantly different according to DeLong’s test. However, the difference between the two AUCs in the test data was not statistically significant according to DeLong’s test. Figure 2C,D show the ROC curves for discriminating the anosmia group from the combined hyposmia-normosmia group using the multivariate regression model (red line) and the PEA bead test-only model (blue dotted line) with the training and test dataset. The AUC values for each case are also presented. The PEA bead test-only model yielded slightly better AUC values of 0.953 and 0.956, compared with AUC values of 0.950 and 0.934 for the multivariate model with the training and test datasets, respectively; however, these differences were not statistically significant according to DeLong’s test. The relatively small difference in AUC values between the training and test datasets for the multivariate model demonstrates the robustness of our model’s fit. Table 3 further presents the 95% confidence intervals for AUCs, sensitivity, and specificity. The maximal sensitivity and specificity values are shown. For discriminating the combined anosmia-hyposmia group from the normosmia group, the multivariate model achieved a sensitivity of 0.83 and a specificity of 0.917 at a cutoff predicted score of >18.5. On the other hand, with the PEA bead test-only model, sensitivity and specificity values of 0.778 and 0.958 were achieved, respectively, at a PEA bead test score cutoff of ≥1. For discriminating the anosmia group from the combined hyposmia-normosmia group, the multivariate model achieved a sensitivity of 1 and a specificity of 0.806 at a cutoff predicted score of >18.5. On the other hand, the PEA bead test-only model achieved a sensitivity of 1 and a specificity of 0.871 at a PEA bead test score cutoff of ≥2 (Table 3).

**Figure 3:**
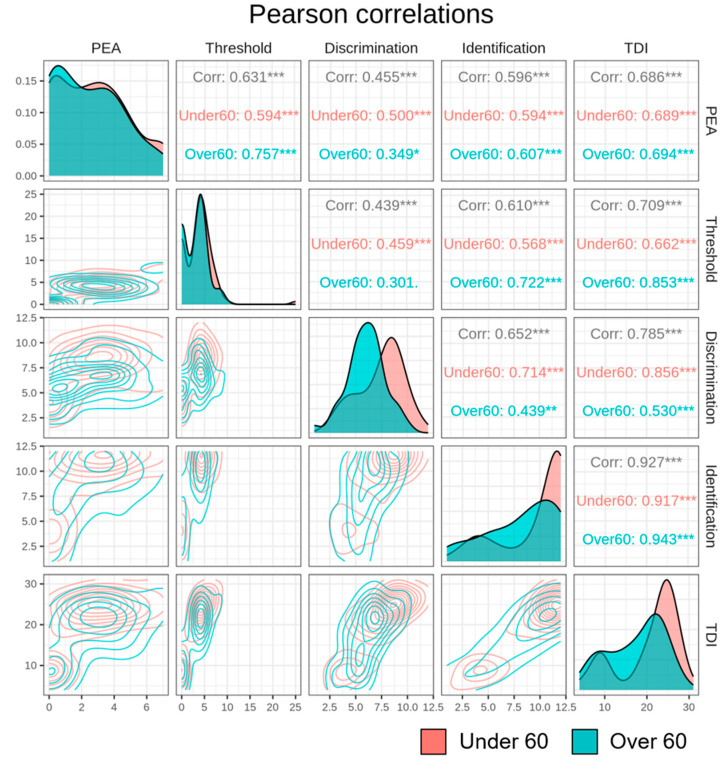
Correlation between the PEA bead test and conventional olfactory function testing with an age-group cutoff of 60 years. The <60-year age group is colored in red, and the ≥60-year age group is colored in green. Top-right triangular components represent Pearson correlations, bottom-left triangular components depict two-dimensional density mapping, and diagonal components represent variable density using kernel density estimation. A contour plot visually represents a 3D surface by plotting constant Z-level lines (contours) on a 2D plane. * *p* ≤ 0.05, ** *p* ≤ 0.01, *** *p* ≤ 0.001.

Finally, we examined whether the relationship between the PEA bead test and YOF test scores changed according to age. Figure 3 shows the Pearson correlations among the PEA bead test, threshold, discrimination, identification, and TDI outcomes of the YOF test with participants categorized as older or younger than 60 years. The diagonal parts in Figure 3 represent the density of each variable. In the case of the PEA bead test variable, the distribution was similar regardless of age group (under or over 60 years), and in the case of the YOF TDI variable, the distribution shape varied by age group. The <60-year age group was unimodal, while the ≥60-year age group was bimodal. In most cases, a significantly strong correlation was found, but in the ≥60-year age group, the PEA bead test and the discrimination subset of the YOF were weakly correlated (*p* < 0.1) (Pearson’s correlation coefficient = 0.349).

## 4. Discussion

We found a significant correlation between the results of the PEA bead test and the YOF test. The results of the PEA bead test were significantly correlated with the results of the threshold, discrimination, and identification subsets of the YOF test, as well as the TDI score. The correlation was statistically significant even in the multivariate analysis considering other clinical variables, such as age, smoking status, underlying diseases, and gustatory dysfunction status. Furthermore, in the regression analysis, the PEA bead-only model showed similar results to the multivariate model, with high sensitivity and specificity for discriminating olfactory dysfunction (anosmia or hyposmia) from normal olfactory function. Therefore, we suggest that the PEA bead test may be a simple and useful screening tool for identifying olfactory dysfunction.

Various conditions have been found to be associated with olfactory dysfunction, and the prevalence of olfactory dysfunction is reportedly increasing. Aging might be among the most important factors, and other major causes include chronic inflammatory conditions of the sinonasal cavity (such as chronic rhinosinusitis with or without nasal polyps), acute infections of the upper respiratory tract, trauma, and neurodegenerative diseases [1,18,19]. Furthermore, after early newspaper reports in many countries, olfactory dysfunction emerged as a potential marker of COVID-19 in March 2020, and a large body of evidence now demonstrates olfactory dysfunction to be among the most common symptoms of COVID-19 infection [4]. Therefore, subjective recognition of olfactory dysfunction has significantly increased, and the necessity of screening for olfactory dysfunction is increasingly important. We suggest that the sniffing bead system might enable routine and rapid olfactory function screening for future potential patients with respiratory diseases, such as COVID-19.

Screening tests are meant to differentiate between health and disease—for example, between normosmia and hyposmia/anosmia [20]. When we evaluated the ROCs, AUCs, and corresponding sensitivity and specificity, the PEA bead test ≥1 category had an AUC of 0.866, with a sensitivity of 0.778 and a specificity of 0.958 (Table 3). This was not inferior to the TDI score of the YOF test, and the specificity was even higher for the PEA bead test variable compared with the YOF test TDI score variable, suggesting that our PEA bead test might be suitable for olfactory dysfunction screening.

Several studies investigating olfactory dysfunction screening have been reported. Most of these studies have been based on identification tests, consisting of several selected odorants that are familiar to the participants [21,22,23]. However, performance on odor identification tests is dependent on verbal abilities, and the results can be influenced by cognitive and language function [1]. The PEA bead test introduced herein is based on threshold testing, which represents a big difference from previously reported screening tests.

Interestingly, we found a consistently strong correlation between the PEA bead test results and YOF test results regardless of age, except in the case of the discrimination subset. Among older participants (≥60 years old), the results of the PEA bead test and discrimination subset of the YOF test were weakly correlated relative to the PEA bead test correlation with the threshold and identification subsets (Figure 3). It has been reported that cortical plasticity is important for associative olfactory memory, and that olfactory memory plays a critical role in odor discrimination [24]. We hypothesized that the impaired olfactory memory function associated with natural aging could explain this weak correlation between the two tests. We suggest that this supports our finding that the PEA bead test, which is not directly associated with olfactory memory function, could be a useful screening tool regardless of age.

The PEA bead test is designed with single-use disposables, and it is free from the risk of infectious respiratory pathogen spread. Various types of odorants can be applied by being packaged into beads. The PEA bead test is simple, and it can be performed in a short duration. These characteristics may enable the PEA bead test to be applied in various clinical settings. However, there were limitations to this study. First, we did not consider qualitative olfactory dysfunction. The symptoms of “parosmia” and “phantosmia” are qualitative dysfunction descriptors. Although parosmic patients do exhibit different brain responses to odors compared with healthy controls [25], these qualitative dysfunction labels are usually based on patients’ subjective symptoms. Therefore, the PEA bead test may show its efficacy as a screening tool only for patients with quantitative dysfunction. Second, the sample size was relatively small. Furthermore, this study was conducted at a single tertiary hospital, and we cannot rule out the possibility that the participants were particularly sensitive to their olfactory function and underwent olfactory function testing enthusiastically (more so than an average member of the general population). Owing to the small sample size of our study, we could not exclude all possible potential factors that affect olfactory function, such as sex and age. Therefore, it is possible that the conclusions of this study would be different if they were based on a larger scale multicenter study. Finally, with the presented PEA test system, older subjects may find it difficult to perform intricate tasks by themselves, and additional ventilation is needed. These mechanical and systematic limitations need to be compensated for to facilitate the clinical application of this system.

## 5. Conclusions

In this study, we introduced the PEA bead test, which is simple, quick, and designed with single-use disposables, minimizing the risk of infectious respiratory pathogen spread. This article summarizes our preliminary results showing that the detection threshold of the PEA bead test was useful for discriminating olfactory dysfunction from normal olfaction. Although our data are preliminary and based on a small sample size, we suggest that the PEA bead test could be applied in various clinical settings as a screening tool for olfactory dysfunction. Future larger scale multicenter studies are warranted to scrutinize our hypothesis.

## Figures and Tables

**Table 1:** Characteristics of enrolled participants.

Variables	Anosmia (*n* = 35)	Hyposmia (*n* = 26)	Normosmia (*n* = 78)	*p* Value
Age	52.71 (18.8)	56.62 (17.95)	43.4 (17.69)	0.004
Sex				0.89
Male	24 (68.6%)	17 (65.4%)	50 (64.1%)	
Female	11 (31.4%)	9 (34.6%)	28 (35.9%)	
Diabetes				0.21
Yes	5 (14.3%)	1 (3.8%)	4 (5.1%)	
No	30 (85.7%)	25 (96.2%)	74 (94.9%)	
Hypertension				0.3
Yes	8 (22.9%)	3 (11.5%)	9 (11.5%)	
No	27 (77.1%)	23 (88.5%)	69 (88.5%)	
Smoking				0.06
Yes	21 (60%)	8 (30.8)	32 (41%)	
No	14 (40%)	18 (69.2%)	46 (59%)	
Gustatory dysfunction				<0.001
Yes	18 (51.4%)	8 (30.8%)	8 (10.3%)	
No	17 (48.6%)	18 (69.2%)	70 (89.7%)	
PEA bead test	0.26 (0.89)	2.38 (1.83)	3.73 (1.76)	<0.001

**Table 2:** Multivariate analysis regarding the relationship between the PEA and YOF tests.

Model	Variable	Beta	SE	95% Confidence Interval		*p* Value
				Lower	Upper	
1	Threshold score, R^2^ = 0.39					
	(Intercept)	1.863	0.485	0.901	2.826	0.000 ***
	Gustatory dysfunction	−1.258	0.676	−2.6	0.084	0.066
	PEA bead test	0.826	0.125	0.578	1.075	0.000 ***
2	Discrimination score, R^2^ = 0.34					
	(Intercept)	7.465	0.347	6.287	8.643	0.000 ***
	Age	−0.021	0.01	−0.042	−0.001	0.044 *
	Diabetes	−2.147	0.832	−3.799	−0.495	0.011 *
	Gustatory dysfunction	−1.359	0.462	−2.277	−0.442	0.004 **
	PEA bead test	0.375	0.086	0.204	0.546	0.000 ***
3	Identification score, R^2^ = 0.42					
	(Intercept)	7.9	0.472	6.962	8.837	0.000 ***
	Hypertension	−1.88	0.764	−3.397	−0.363	0.016 *
	Gustatory dysfunction	−1.851	0.64	−3.122	−0.581	0.005 **
	PEA bead test	0.725	0.119	0.49	0.96	0.000 ***
4	TDI score, R^2^ = 0.55					
	(Intercept)	18.705	1.547	15.633	21.777	0.000 ***
	Age	−0.06	0.027	−0.112	−0.006	0.030 *
	Gustatory dysfunction	−4.172	1.209	−6.574	−1.77	0.001 ***
	PEA bead test	1.818	0.224	1.372	2.264	0.000 ***

Abbreviation: PEA, 2-phenylethyl alcohol. * *p* ≤ 0.05, ** *p* ≤ 0.01, *** *p* ≤ 0.001.

**Table 3:** AUC, sensitivity, and specificity analysis of the PEA bead test for olfactory dysfunction screening.

	Model	Cutoff Value	AUC	Sensitivity	Specificity
Combined anosmia-hyposmia vs. normosmia	Multivariate	TDI > 18.5	0.898 (0.802–0.994)	0.83	0.917
	PEA bead test	PEA ≥ 1	0.866 (0.739–0.992)	0.778	0.958
Anosmia vs. combined hyposmia-normosmia)	Multivariate	TDI > 18.5	0.934 (0.862–1)	1	0.806
	PEA bead test	PEA ≥ 2	0.956 (0.902–1)	1	0.871

Abbreviations: AUC, area under the receiver operating characteristic curve; PEA, 2-phenylethyl alcohol; TDI, threshold-discrimination-identification score.

## Data Availability

This study’s data are available from the corresponding author upon reasonable request. The data are not publicly available.

## Supplementary Materials

The following supporting information can be downloaded at https://www.mdpi.com/article/10.3390/life13102074/s1, Figure S1: Image of the PEA sniffing bead test. (A) The sniffing bead system is composed of odorant beads, a handpiece, and a lid. (B) Beads packed with various concentrations of 2-phenylethyl alcohol (PEA) and beads packed with distilled water were used.
