# Supplementary material for: The PEA Bead Test as a Screening Tool for Olfactory Dysfunction: A Preliminary Study
Source: Life (Basel). 2023 Oct 17;13(10):2074. doi: 10.3390/life13102074 (PMC10608016; doi:10.3390/life13102074)
Supplement: Supplementary file 1 [file life-13-02074-s001.zip › life-2631396-supplementary.pdf]

Supplementary Material

The PEA Bead Test as a Screening Tool for Olfactory Dysfunction: A Preliminary Study

Kyung Soo Kim [1], Il-Youp Kwak [2] and Hyun Jin Min [1,*]

[1] Department of Otorhinolaryngology-Head and Neck Surgery, College of Medicine, Chung-Ang University, Seoul 06973, Republic of Korea; entkks@cau.ac.kr
[2] Department of Applied Statistics, Chung-Ang University, 224-1 Heukseok-dong, Dongjak-gu,
Seoul 06973, Republic of Korea; ikwak2@cau.ac.kr
* Correspondence: jjinient@cau.ac.kr; Tel.: +82-2-6299-1765

Figure S1

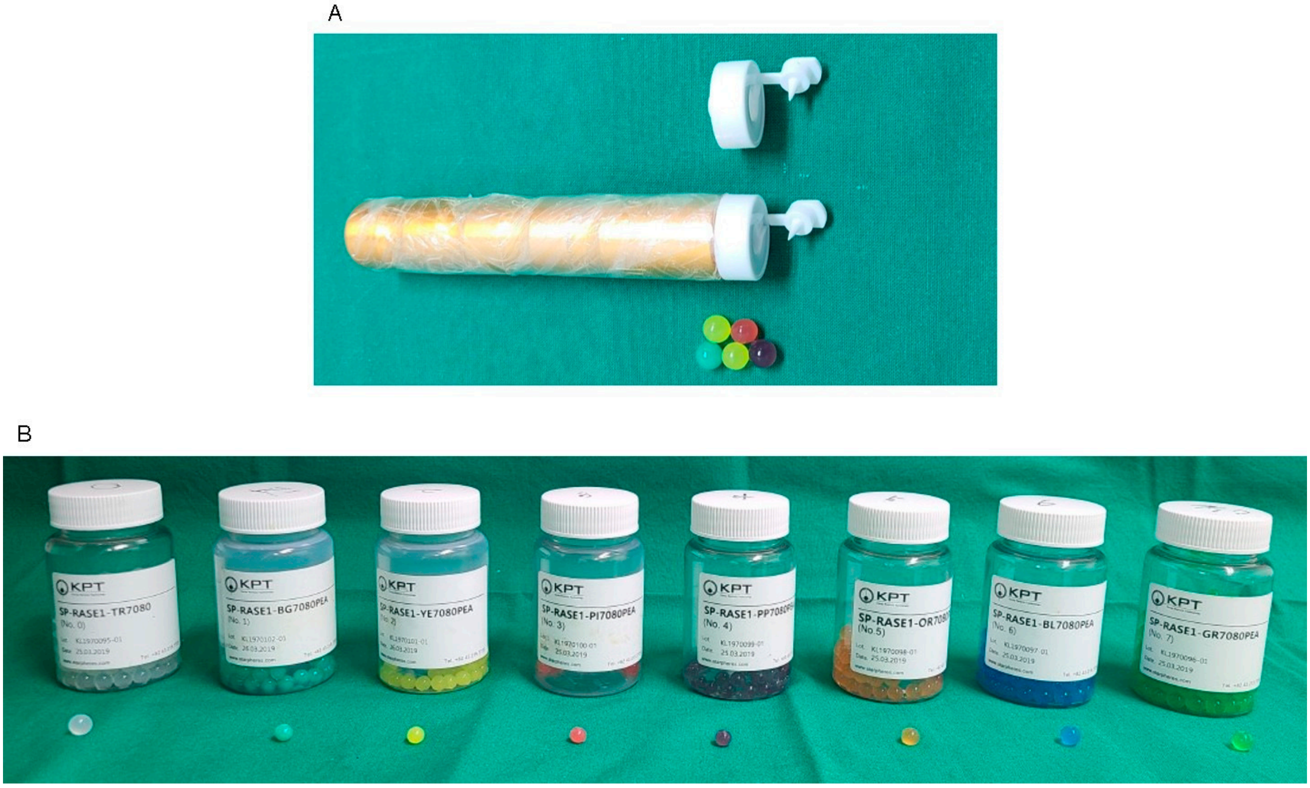

Figure S1. Image of the PEA sniffing bead test. (A) The sniffing bead system is composed of odorant beads, a handpiece, and a lid. (B) Beads packed with various concentrations of 2-phenylethyl alcohol (PEA) and beads packed with distilled water were used.
